# Evaluation of different OCT systems in quantitative imaging of human Schlemm's canal

**DOI:** 10.1038/s41598-022-05410-9

**Published:** 2022-01-26

**Authors:** Xuan Wu, Bingyao Tan, Jinyuan Gan, Adeline R. Lam, Yibing Chen, Xinyu Liu, Jacqueline Chua, Damon W. K. Wong, Marcus Ang, Leopold Schmetterer, Xinwen Yao

**Affiliations:** 1 Singapore Eye Research Institute, Singapore National Eye Centre, Singapore, Singapore; 2 SERI-NTU Advanced Ocular Engineering (STANCE), Singapore, Singapore; 3 School of Chemical and Biomedical Engineering, Nanyang Technological University, Singapore, Singapore; 4 Academic Clinical Program, Duke-NUS Medical School, Singapore, Singapore; 5 Department of Clinical Pharmacology, Medical University of Vienna, Wien, Austria; 6 Center for Medical Physics and Biomedical Engineering, Medical University of Vienna, Wien, Austria; 7 Institute of Molecular and Clinical Ophthalmology, Basel, Switzerland

**Keywords:** Three-dimensional imaging, Diagnostic markers

## Abstract

We examined the performance of human Schlemm’s canal (SC) imaging using different OCT devices: CIRRUS 5000 (840 nm, spectral-domain (SD)-OCT), PLEX Elite 9000 (1060 nm, swept-source (SS)-OCT) and CASIA SS-1000 (1310 nm, SS-OCT), and analyzed potential impact factors on visualization and the quantitative assessment of SC morphology in a pilot study. Ten healthy subjects were imaged using three OCT devices by a single experienced operator on the same day. Each eye underwent two cubic scans by each device, one on nasal and the other on temporal quadrant. The B-scan showing the largest SC was manually selected for processing. Four quantitative metrics, including one morphological metric as cross-sectional area (CSA), and three performance metrics as contrast, continuity, and coverage, were derived from the datasets. Repeated-measures ANOVA was used to investigate the difference between these parameters from the three devices (*P* < 0.05). We found the CSA measured from CIRRUS was significantly larger than PLEX, followed by CASIA. The contrast was highest in CIRRUS, followed by PLEX and CASIA. The coverage was also higher in CIRRUS as compared to PLEX and CASIA. No significant difference was seen in the continuity from the three devices. In summary, we showed the measurements from the three devices were not interchangeable.

## Introduction

Schlemm’s canal (SC) is a tube-like structure located at the inner part of the corneoscleral junction which goes around the cornea. It plays an important role in intraocular pressure (IOP) regulation by draining the excess aqueous humor from the anterior chamber of the eye to the episcleral veins^1^. SC is closely associated with glaucoma, the second leading cause of blindness worldwide^2^. Anatomical alterations in Schlemm’s canal are linked to elevated IOP, the most important modifiable risk factor for such disease^3,4^. Studies have shown that narrowing or progressive collapse of the SC observed in glaucomatous eyes may be responsible for the increased aqueous outflow resistance and the elevated IOP^5,6^. SC dimensions are correlated with the presence of primary open angle glaucoma (POAG)^1,7–11^. Surgical procedures, such as canaloplasty^12^, viscocanalostomy^13–15^, and micro-stent implantation^16–18^, were developed targeting on mechanical dilation of the SC to lower the IOP, whilst therapeutic interventions that facilitate SC dilation have also been investigated to achieve similar results^19^. Moreover, mounting evidence pointed that the flow to the aqueous veins is pulsatile due to the change in SC dimensions^20,21^. Accurate visualization and real-time assessment of SC dimensions via in vivo imaging will enable better understanding of the SC and its role in regulating the flow in the aqueous humor outflow (AHO) facility.

Optical coherence tomography (OCT) is a non-invasive imaging technique that offers high-resolution and three-dimensional (3D) visualization of the SC structure and can be applied to a wide variety of clinical problems at the anterior segment (AS)^22,23^. Several studies have reported on in vivo SC quantifications using spectral-domain (SD) AS OCT operated at 850 nm spectral window^9,24–29^, but most of the studies shared the common problem of poor SC discerning ability due to the shadowing artifact casted by superficial vessels as well as blurring effect induced by eye motion. This is mainly due to two reasons. First, commercial SD OCT systems adopted in these studies relied on the line-scan camera with an A-scan speed limited to 70 kHz. At this speed, one single volumetric scan may take at least several seconds to complete, and the image quality will be inevitably deteriorated by motion artifacts. Second, photons at a shorter wavelength experience higher scattering loss when travelling deeper into the tissue. Previous study suggested that the SC may reside as deep as 1.1 mm under the limbus area. At this depth, the backscattered photons from the SC may suffer from greater attenuation which leads to a lower signal-to-noise ratio of the SC structure. Moreover, structures that reside on top of the SC, such as the epi-scleral and intra-scleral vessels as well as the pigmentation in the limbus, may further attenuate the signal. On the contrary, swept-source (SS)-OCT utilizes light sources at longer wavelengths (1060 nm or 1310 nm) that allow for better signal penetrability to detect deeper structures. Combined with the longer imaging depth and alleviated sensitivity roll-off effect, it facilitates the visualization of deep tissue structures such as SC, iridocorneal angle, sclera spur, and trabecular meshwork (TM). Furthermore, the swept sources can offer a higher A-scan speed which may substantially reduce the motion artifacts. Nevertheless, the resolution offered by SS-OCT is usually inferior to its SD counterpart, and it is unclear whether it will affect the quantification of SC. To date, it is still unclear what optical wavelength window is optimal for OCT imaging of SC, and a quantitative comparison of SD- and SS-OCT on SC imaging performance is still lacking.

In this study, we quantitatively compared the performance of three commercial OCT systems, Zeiss CIRRUS 5000 (CIRRUS, 840 nm, SD-OCT), Zeiss PLEX Elite 9000 (PLEX, 1060 nm, SS-OCT) and Tomey CASIA SS-1000 (CASIA, 1310 nm, SS-OCT), in SC imaging and analyzed the potential impact factors. The cross-sectional area (CSA) of SC was extracted via manual segmentation and compared among different devices. Besides, we proposed three performance metrics, namely the contrast, the coverage, and the continuity of the SC, to evaluate the discernibility of SC in these three devices.

## Results

The demographics and clinical characteristics of these participants are summarized in Table 1. It shows that the mean age of the subject was 33.1 years, with 40% male and 80% Chinese. Their mean spherical equivalent was -4.24. For each patient, one volume was randomly picked for the two graders to assess the continuity and coverage (see Supplementary Fig. S1 online for illustration). The inter-rater agreement levels for the two graders were excellent on continuity (CIRRUS: ICC = 0.98; PLEX: ICC = 0.97; CASIA: ICC = 0.97) and coverage measurements for all devices (CIRRUS: ICC = 0.95; PLEX: ICC = 0.96; CASIA: ICC = 0.95). Hence their assessments were averaged for calculation of the quantitative metrics.

**Table 1:** Clinical and ocular characteristics of participants (*N* = 10 participants).

Characteristics	Mean (SD), *n* (%)
**Clinical characteristics**	
Age (years)	33.1 (6.54)
Sex, male	4, 40%
Ethnicity, Chinese	8, 80%
**Ocular characteristics**	
Imaged eye	20
Spherical equivalent	-4.24 (2.67)

*SD* standard deviation.

**Figure 1:**
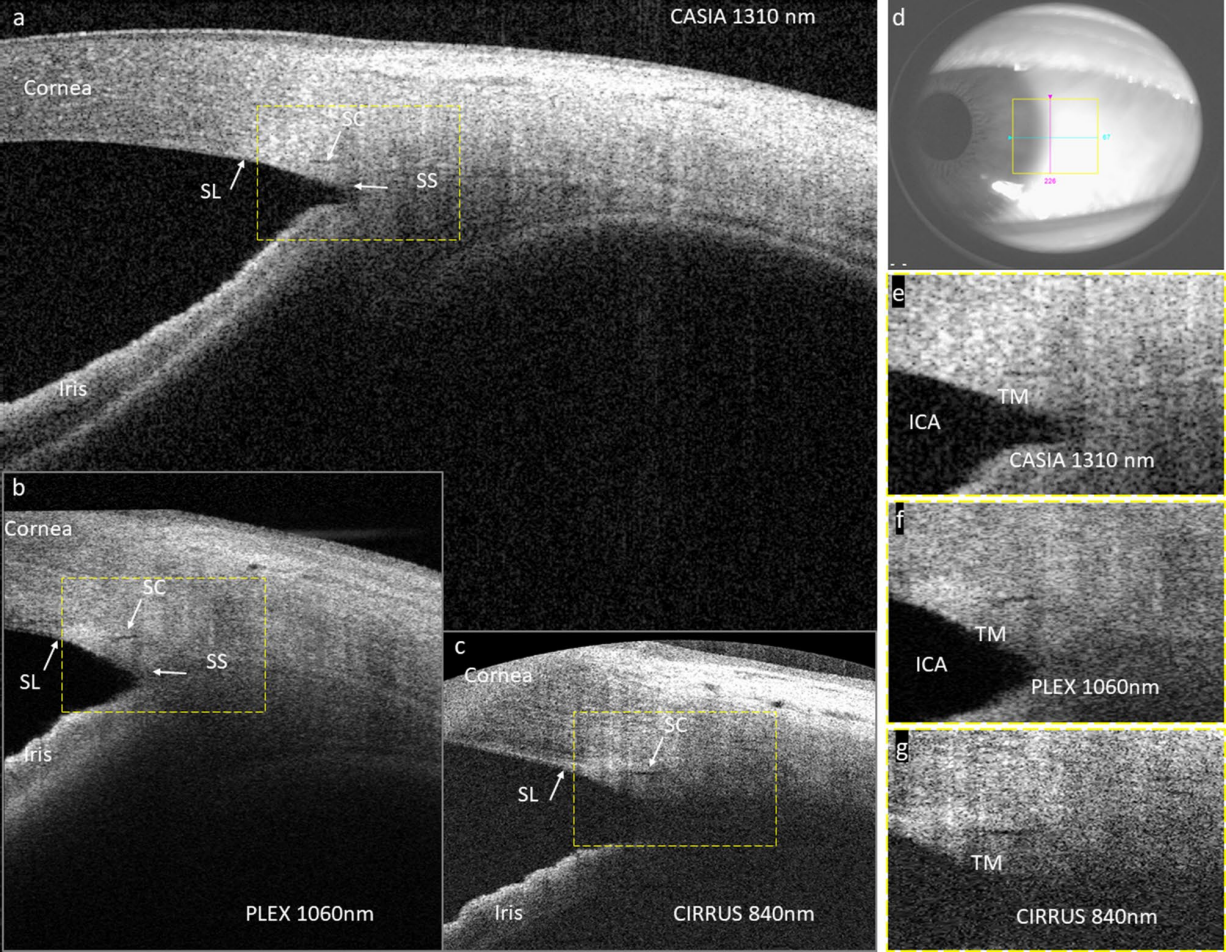
(**a**)–(**c**) Selected OCT B-scans of SC obtained by CASIA, PLEX, and CIRRUS, respectively, taken at the same quadrant of the same eye. The regions-of-interest (ROIs) of Schlemm’s canal are marked by yellow dashed rectangles. (**d**) White-light camera image showing the acquisition location. (**e**)–(**g**) Zoom-in views of the SC ROIs in CASIA, PLEX, and CIRRUS images, respectively. ICA: iridocorneal angle; SC: Schlemm’s canal; SL: Schwalbe’s line; SS: scleral spur; TM: trabecular meshwork.

Figure 1 offers a visual comparison between SC images acquired on the same quadrant of one healthy eye from the three devices. The contrast of SC was seen best in CIRRUS compared to PLEX and CASIA images. The sharpness of PLEX image was lower compared to CIRRUS and CASIA images because it was an average of four images acquired at the same location without motion compensation. The penetration depth in tissue was seen shallowest in CIRRUS and deepest in CASIA. Deep tissue features such as scleral spur and iridocorneal angle were delineated in both PLEX and CASIA but not in CIRRUS. Moreover, the shadowing effect, while strongest in CIRRUS, was most alleviated in CASIA.

Table 2 summarises the morphometric as well as performance metrics of SC measured from CIRRUS, PLEX, and CASIA. The CSA was largest measured from CIRRUS (9552.78 ± 1406.03µm^2^), followed by PLEX (8643.46 ± 1573.69µm^2^), and smallest by CASIA (7890.57 ± 1246.92µm^2^). The contrast was seen significantly different in three devices (*p* < 0.001), with the highest in CIRRUS (0.60 ± 0.05), mediocre in PLEX (0.52 ± 0.06), and lowest in CASIA (0.41 ± 0.05). In addition, CIRRUS exhibited the best coverage (87.6% ± 11.8%), followed by PLEX (76.7% ± 14.8%), and CASIA (71.3% ± 17.6%). On the other hand, the continuity of SC visualization varied greatly within the datasets. The mean continuity was seen highest in CIRRUS (73.5% ± 23.2%), followed by PLEX (65.3% ± 21.7%) and CASIA (60.0% ± 21.7%), although no significant difference was found between three devices.

**Table 2:** Quantitative metrics of SC from three AS-OCT devices.

OCT device	CIRRUS (*N* = 120)	PLEX (*N* = 120)	CASIA (*N* = 120)	*P*-value
Cross sectional area (SD), µm^2^	9552.78 (1406.03)	8643.46 (1573.69)	7890.57 (1246.92)	** < 0.001**
Mean difference, µm^2^ CIRRUS – PLEX (95% CI)	909.32 (359.47, 1459.17)			**0.011**
Mean difference, µm^2^ CIRRUS – CASIA (95% CI)	1662.21 (1147.86, 2176.57)			** < 0.001**
Mean difference, µm^2^ PLEX – CASIA (95% CI)	752.89 (224.76, 1281.03)			**0.035**
Contrast (SD)	0.60 (0.05)	0.52 (0.06)	0.41 (0.05)	**< 0.001**
Mean difference CIRRUS – PLEX (95% CI)	0.08 (0.06,0.10)			** < 0.001**
Mean difference CIRRUS – CASIA (95% CI)	0.19 (0.17,0.21)			** < 0.001**
Mean difference PLEX – CASIA (95% CI)	0.11 (0.09,0.13)			** < 0.001**
Coverage (SD), %	87.6 (11.8)	76.7 (14.8)	71.3 (17.6)	** < 0.001**
Mean difference, % CIRRUS – PLEX (95% CI)	10.84 (5.47, 16.22)			**0.015**
Mean difference, % CIRRUS – CASIA (95% CI)	16.23 (9.53, 22.93)			**< 0.001**
Mean difference, % PLEX – CASIA (95% CI)	5.39 (0.22, 10.56)			0.29
Continuity (SD), %	73.5 (23.2)	65.3 (21.7)	60.0 (21.7)	> 0.05

Significant values are in bold.

*SD* standard deviation; *CI* confidential interval.

**Figure 2:**
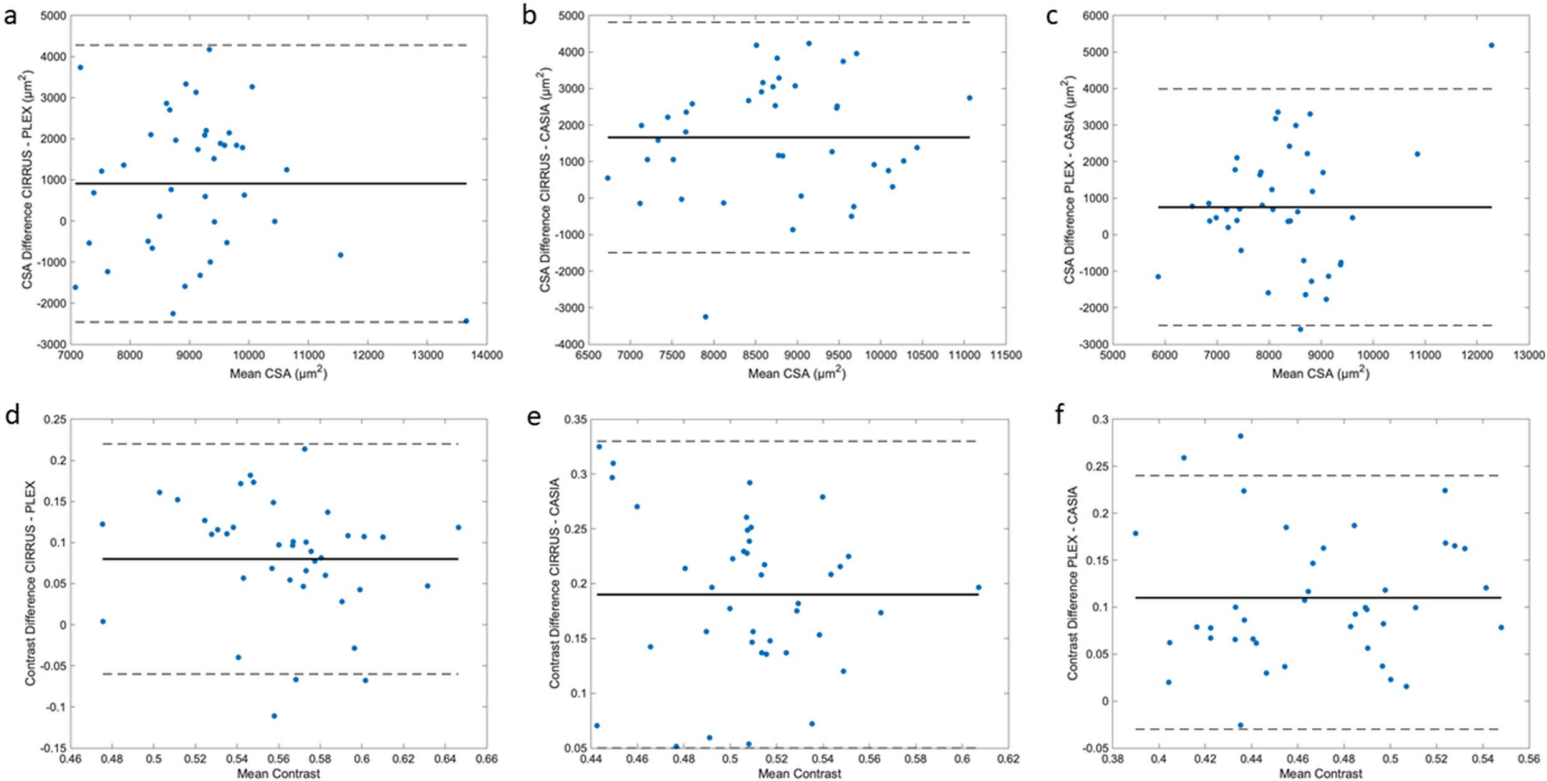
(**a**)–(**c**) Bland–Altman plots of CSA measurements from CIRRUS, PLEX and CASIA. (**d**)–(**f**) Bland–Altman plots of contrast measurements from CIRRUS, PLEX and CASIA.

Figure 2a–c shows the Bland–Altman plots comparing the CSA measured from the three devices. The difference was greatest between the measurements from CIRRUS and CASIA (Δ = 1662.21µm^2^, *p* < 0.001), and the difference was significant between CIRRUS and PLEX (Δ = 909.32µm^2^, *p* = 0.011), as well as PLEX and CASIA (Δ = 752.89µm^2^, *p* = 0.035). Figure 2e, f shows the Bland–Altman plots comparing the contrast measured from the three devices. Both CIRRUS and PLEX offers better contrast compared to CASIA (mean difference CIRRUS–CASIA = 0.19; PLEX–CASIA = 0.11; CIRRUS–CASIA = 0.08; *p* < 0.001).

## Discussion

In this study, we investigated the performance of three different OCT devices on SC visualization by comparing both morphological and performance metrics. Overall, we observed significant differences exhibited in the CSA derived from the three devices. Specifically, CIRRUS yielded significantly higher CSA measurement of SC compared to PLEX and CASIA, mainly due to its higher optical resolution and higher sampling density. The SC CSA characterization was performed by the subjective “search and measure” method, where one single B-scan showing the largest SC was manually picked from the respective OCT volumes and CSA was measured from that B-scan only. Although the CSA may be likely overestimated by this method, it was considered representative of the comprehensive assessment result of the CSA using all B-scans^24^. Hence, it is safe to attribute the difference seen between different devices to the specifications of the devices, rather than the subjective characterization method.

The performance metrics were designed to assess the discernability of SC from the OCT devices: contrast measures the local SNR of the SC in each image; coverage is a measure to assess the portion of SC that can be successfully visualized within an OCT volume; continuity shows how well the continuous variation in SC size was captured by each device. CIRRUS offered the best 3D visualization performance in terms of contrast and coverage. The contrast advantage of CIRRUS can be attributed to the higher sensitivity, the stronger backscattering, as well as the superior optical resolution associated with the shorter wavelength. The coverage and continuity will be affected by not only the aforementioned factors but also the scan speed and sampling density in the slow scan direction. It can be seen from the results that none of them offered 100% continuous coverage of SC within one volume although SC was supposed to remain open in healthy eyes. It illustrates the challenges of SC visualization using OCT, which include 1) shadowing obscureness from the superficial tissues like epi- and intra- scleral blood vessels and the conjunctiva; 2) deterioration from eye movement under slow volumetric scan speed; 3) reduced SNR and insufficient resolution at the iridocorneal recess where SC resides. As seen in Fig. 1, although CIRRUS provided the highest contrast of SC, it has the lowest signal penetration in tissue and deep tissue features, such as scleral spur and iridocorneal angle, which cannot be fully delineated. Moreover, the shadowing effect was most severe in CIRRUS. These factors make the identification of SC more difficult in CIRRUS images. Conversely, PLEX and CASIA were able to delineate most of the key anatomical features needed to identify the SC, while manifesting less shadowing effect compared to CIRRUS. This observation demonstrates the benefit of adopting longer wavelengths in SC imaging. In terms of suppression of eye movement, PLEX was supposed to outperform the other two devices with an A-scan speed three times higher than that of CIRRUS and CASIA. Nevertheless, PLEX only employs OCT-A protocols where each structural OCT B-scan is a result of averaging several repeated B-scans taken at one slow-scan location. Considering that the size of SC may be changing over time to produce a pulsatile flow and eye motion is not compensated during image acquisition, this averaging process may wash out details of SC, leading to a reduced contrast as well as less coverage and continuity in SC visualization, and undermining the advantage brought by the scan speed. We recently investigated a mega-Hertz SS-OCT device at 1060 nm for SC visualization and showed that it was possible to suppress the motion artifact when the volumetric acquisition was done within one second^30^. We also found full coverage of SC within a single volume and successfully segmented the SC from each of the B-scans. These results suggested that, although the SNR plays an important role in the delineation of SC, the acquisition speed as well as motion suppression seem to have major impacts on SC visualization in general^31^. It would be beneficial for studies of SC provided that more flexibility is offered by the manufacturer in terms of image acquisition protocols.

There are also some limitations of this study. First, due to the difference in sampling density along the slow scan direction, the B-scans that were chosen for SC comparison might not be well registered for all three devices. Second, the images from PLEX were not corrected for refractive error and neither was its scan protocol optimized for AS-OCT structural imaging. Refractive correction may affect surface curvature measurement as well as thickness measurement of various anatomical structures in the anterior segment^32^, especially for structures under curved surfaces. By noting this issue, we intentionally designed the imaging protocol so that the patient would need to look sideway when SC images were taken, and that the SC was positioned at the center of the image where the light incidents normally into the sclera. Given that the cross section of SC is relatively small, it is safe to assume that the refractive correction only affects the axial depth but not the other morphological parameters. Still, the quantitative metrics of PLEX might be not representative for SC characterization at 1060 nm SS-OCT. Third, the iris was not restricted during the imaging session, and therefore the morphology of SC might be affected by the ambient light. Fourth, the refractive index of sclera was generally unknown and therefore the absolute values of CSA measurement may not be accurate. Finally, as a pilot study, we only incorporated a small sample size (10 subjects) with a narrow age range (26–42 years), and only healthy participants were included. Our morphologic measurements of SC may therefore not be representative enough for older and glaucoma individuals.

In conclusion, we quantitatively compared the performance of three commercial OCT systems Cirrus 5000 with 840 nm, PLEX Elite 9000 with 1060 nm and Casia SS-1000 with 1310 nm in SC visualization and measurements and showed that the measurements from these 3 devices were not interchangeable. The wavelength and resolution of the anterior segment OCT system may affect the visualization as well as the quantitative assessment of SC morphology. The 840 nm SD-OCT showed the best performance in SC visualization in terms of contrast and coverage, while the 1310 nm SS-OCT excelled in penetration depth. The 1060 nm SS-OCT showed a good balance in penetration depth and contrast.

## Methods

### Study participants

In this study, 10 healthy volunteers aged 26 and above with no history of systemic or ocular diseases were recruited for SC evaluation from July 2020 to September 2020 at the Singapore Eye Research Institute. All subjects were screened for suitability before recruitment. Written informed consents were obtained from all participants in accordance with the Declaration of Helsinki. The experimental protocol as well as all procedures performed were approved and in adherence with the ethical standards of the SingHealth Centralised Institutional Review Board (IRB)^33^.

### Ocular examination

Detailed interviewer-administered questionnaire was used to screen for any chronic medical history (e.g. diabetes, hypertension, high cholesterol) and ocular history (e.g. glaucoma, retinopathies or any surgery and laser treatment). Before undergoing AS-OCT imaging, participants were then assessed for their refractive error using an autorefractor (Canon RK-5 Autorefractor Keratometer; Canon Inc., Tokyo, Japan), as well as their intra-ocular pressure. Fundus photographs further documented the absence of any ocular diseases.

### Anterior segment OCT imaging

Anterior segment OCT imaging was performed by three different OCT devices: CIRRUS (ZEISS CIRRUS 5000, Carl Zeiss Meditec, USA), PLEX (ZEISS Plex Elite 9000, Carl Zeiss Meditec, USA), and CASIA (CASIA Corneal/Anterior Segment OCT SS-1000, Tomey Corporation, Japan), with a summary of the system specifications presented in Table 3. CIRRUS is an SD-OCT system with a central wavelength of 840 nm and a speed of 27,000 A-scans/s. The axial and lateral resolutions are 5 µm and 20 µm, respectively. The scanning protocol (Anterior Segment 512 × 128) covers a 4 mm × 4 mm area with 128 B-scans, of which each B-scan has 512 A-scans. PLEX is an SS-OCT prototype system that was designated for OCT angiography of the posterior pole, with a central wavelength of 1060 nm and a speed of 100,000 A-scans/s. A 20-diptor(20D) anterior segment lens was employed to convert it to AS imaging mode. The axial and lateral resolutions in tissue are 6.3 µm and 21 µm, respectively. The 3 mm × 3 mm OCT-A protocol was adopted, which effectively covers a 4 mm × 4 mm area under the AS imaging mode. Each volume is composed of 300 B-scans (4-time repetition for one location) with 300 A-scans in each B-scan. Similarly, CASIA is also an SS-OCT device, but is designated for AS imaging. It has a central wavelength of 1310 nm and operates at a speed of 30,000 A-scans/s. The axial and lateral resolutions in tissue are 10 µm and 30 µm, respectively. The scanning protocol of “8 mm × 4 mm Angle HD” covers an area of 8 mm × 4 mm with 512 A-scans per B-scan and 64 B-scans per volume.

**Table 3:** Summary of AS-OCT devices.

OCT device	CIRRUS	PLEX*	CASIA
Central wavelength (nm)	840	1060	1310
Imaging speed (A-scans/s)	27,000	100,000	30,000
Axial resolution in tissue (µm)	5	6.3	10
Digital pixel number	1213	1536	900
Image depth (mm)	2	3	6
Lateral resolution in air^†^ (µm)	20	21	30
Scan area (mm × mm)	4 × 4	4 × 4	8 × 4
Sampling density (fast axis)	512	300 (× 4)	512
Sampling interval (fast axis) (µm)	7.8	13.3	15.6
Sampling density (slow axis)	128	300	64
Sampling interval (slow axis) (µm)	31.3	13.3	62.5

*PLEX was operated with an AS attachment of 20D with a focal length of 50 mm. The 3 × 3 AngioPLEX scan conducts 4-time repetition of B-scan for one location.

^†^Lateral resolution of PLEX was estimated using the formula $r=0.8\cdot \lambda \cdot f/D$, where the beam diameter *D* estimated to be 2 mm.

For each device, both eyes of the subjects underwent two cubic scans, one on the nasal quadrant and the other temporal, on the same day by a single experienced operator. An external fixation target was introduced to allow specular incidence of light onto the limbal area above the SC. Landmarks such as epi-scleral vessels can be identified from the white light images of the respective device and served as assignment guidance for volume registration when navigating across different devices. All the B-scans were directly exported from the respective OCT devices for processing.

### Image processing and Schlemm’s canal quantification

Schlemm’s canal can be identified from the OCT B-scans as a curvilinear lucent area, extended from the SS to the anterior tip of the TM located at the end of the Descemet's membrane, while external to the TM^34^.

Each dataset was rescaled and resampled to the same dimension of 4 mm by 4 mm laterally and 2 mm axially, with an isotropic pixel size of 1.95 µm using bilinear interpolation. One single B-scan showing the largest SC in each volume was chosen (XW) for processing^24^. The selected B-scans were registered for all 3 devices (see Fig. 1a–c). A 230-by-100 pixels rectangular region of interest (ROI) was then manually selected to restrict the area that contains SC. Two trained graders (JG, ARL) independently marked out the SC lumen structures from the ROIs. The cross-sectional area (CSA), treated as the area (in µm^2^) of the individual segmentation mask by multiplying the total pixel number within the region and the single pixel area, was automatically extracted using ImageJ (National Institutes of Health, Bethesda, Maryland, USA), and then averaged to generate the final measurement for SC in each image. The performance metrics, including the contrast, the coverage, as well as the continuity, were used to evaluate the performance of three OCT devices in their discernability of the SC structure. The contrast was defined by Michelson contrast as (I_max_-I_min_)/(I_max_ + I_min_), where I_max_ is the intensity value of the brightest intensity value outside the segmented SC boundary within the ROI, and I_min_ is the averaged intensity value of the area enclosed by a boundary that was 50 pixels inferior to the SC boundary (see Supplementary Fig. S1 online). It was generated using MATLAB (MathWorks, Natick, MA, USA). The coverage was defined as the fraction of B-scans with clear SC delineation over all B-scans of one volume, and the continuity as the fraction of the maximum continuous B-scans with clear SC delineation over all B-scans of the volume (see Supplementary Fig. S1 online). The two graders independently assessed each volume and determined on these two metrics.

### Statistical analysis

Intraclass correlation coefficients (ICCs) together with 95% confidence intervals was performed to assess the agreement between two graders on qualitative assessment of continuity and coverage, where ICC values less than 0.5, between 0.5 and 0.75, between 0.75 and 0.90, and greater than 0.90 indicate poor, moderate, good, and excellent agreement, respectively. Bland–Altman plots were used for visual representation of CSA and contrast measurements between different devices. Repeated measure ANOVA with Tukey’s post-hoc test was used for quantitative comparison between three devices. Only metrics that manifested statistically significant difference was reported. A *P*-value less than 0.05 was considered significant. All statistical analysis was performed using Stata version 15, except repeated measures ANOVA using IBM SPSS commercial analytical software (IBM SPSS statistic 23). The Bland–Altman plots were generated in MATLAB.

## Supplementary Information

Supplementary Information.
