# Supplementary material for: Evaluation of different OCT systems in quantitative imaging of human Schlemm's canal
Source: Sci Rep. 2022 Jan 26;12:1400. doi: 10.1038/s41598-022-05410-9 (PMC8792050; doi:10.1038/s41598-022-05410-9)
Supplement: Supplementary file 1 — Supplementary Information. [file 41598_2022_5410_MOESM1_ESM.docx]

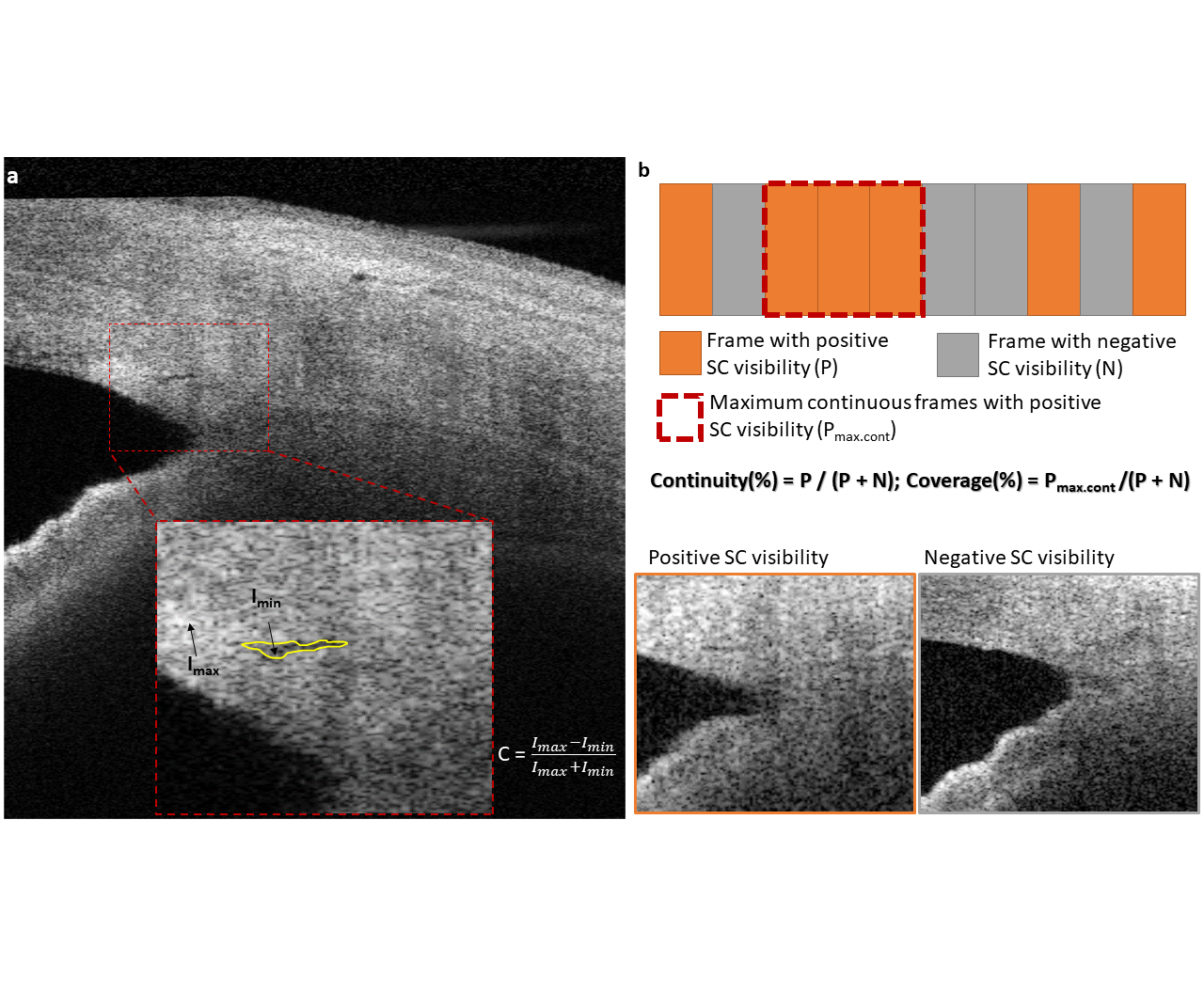
Supplementary Figure S1. Illustration of the performance metrics. (a) Illustration of the contrast calculation. C: contrast; I_min_: minimum intensity within the marked region; I_max_: maximum intensity within the ROI. (b) Illustration of the definition of continuity and coverage.
